# Pathological study of a traumatic anthropogenic injury in the skeleton of a spiny butterfly ray (*Gymnura altavela*)

**DOI:** 10.3389/fvets.2024.1452659

**Published:** 2024-10-24

**Authors:** Gustavo Montero-Hernández, María José Caballero, Ángel Curros-Moreno, Cristian M. Suárez-Santana, Miguel A. Rivero, Lucía Caballero-Hernández, Mario Encinoso, Antonio Fernández, Ayoze Castro-Alonso

**Affiliations:** ^1^Institute of Animal Health and Food Safety (IUSA), College of Veterinary Medicine, University of Las Palmas de Gran Canaria, Las Palmas, Spain; ^2^Poema del Mar Aquarium, Loro Parque Fundación, Las Palmas, Spain; ^3^Veterinary Hospital, College of Veterinary Medicine, University of Las Palmas de Gran Canaria, Las Palmas, Spain

**Keywords:** *Gymnura altavela*, elasmobranch, clinical presentation, computed tomography, veterinary pathology, cartilage

## Abstract

**Introduction:**

External injuries in elasmobranchs are frequent findings, either due to inter- or intraspecific interactions or as a result of interaction with human activities. However, the resilience of these species to traumatic injury remains poorly understood. This work provides an insight into the clinical presentation, diagnostic imaging, and pathological features of a severe traumatic injury to the cartilaginous skeleton of a spiny butterfly ray (*Gymnura altavela*).

**Methods:**

An adult female was found lethargic in the bottom of the coast of Gran Canaria, with an external incised-contused traumatic lesion of 2 cm diameter in the scapulocoracoid cartilage. It was captured and transferred to the Poema del Mar Aquarium for its clinical evaluation and treatment. Despite these efforts, the animal eventually died and was transfer to the Institute of Animal Health and Food Safety (IUSA) for its pathological diagnosis, including a Computed Tomography (CT) study and necropsy.

**Results:**

The animal presented a marked reduction in hematocrit and hepatosomatic index due a chronic debilitation process. The CT scan revealed a destructive lesion with irregular margins at the level of the right scapulocoracoid cartilage. The main pathological findings were the disorganization of the tesserae layer, appearing as whitish square to rectangular geometric pieces separated from the cartilaginous core. Histologically, these pieces of tesserae were separated from the unmineralized cartilage core and displaced from the adjacent perichondrium, where inflammatory cells infiltrate. Edema and hemorrhages were also observed.

**Conclusions:**

This study reports the first comprehensive description of skeleton trauma in a spiny butterfly ray, including the clinical presentation, diagnostic imaging and the anatomopathological features.

## 1 Introduction

The situation of elasmobranchs is critical, being considered as one of the most endangered groups of animals on the planet (1, 2). Anthropogenic causes, primarily fishing interactions, stand out as the foremost threats and causes of death in sharks and rays (1, 3, 4). Located in the Atlantic Ocean, the Canary Islands are an archipelago of volcanic origin that constitutes a Spanish autonomous community situated off the northwest coast of Africa. Comprising eight inhabited islands (Gran Canaria, Tenerife, Fuerteventura, Lanzarote, La Palma, La Gomera, El Hierro, and La Graciosa) alongside various uninhabited islets. The distinctive environmental characteristics of this archipelago result in a unique biodiversity, with a high number of endemic species (5–7). This region represents a stronghold for a wide variety of endangered elasmobranchs species, where they form stable populations and have nurseries in locations with high human presence (8–13). The spiny butterfly ray (*Gymnura altavela*) is among the most frequently observed elasmobranchs in the Canary Islands (10). This ray species belongs to the family *Gymnuridae* and is distributed throughout the coastal waters of the Atlantic Ocean, Mediterranean Sea, and Black Sea, where it primarily inhabits sandy substrates ranging from the seashore to depths of 150 meters (14). It is classified as Critically Endangered in Europe and Endangered worldwide in the International Union for Conservation of Nature (IUCN) Red List due to declining populations mainly because of fishing pressure and habitat destruction (15). The vast presence of tourism along the coasts of the Canary Islands, raises significant interest in studying the potential threats by human actions to the marine environment.

Particularly, traumatic injuries in wild elasmobranchs often arise from both intraspecific and interspecific encounters, or because of interactions with human activity (16–24). The remarkable capacity and rapidity of external wound healing in chondrichthyans are widely acknowledged (25–29). For instance, severe injuries consistent with vessel collisions have been documented in a great white shark (*Carcharodon carcharias*) (30), a reef manta ray (*Mobula alfredi*) (31), and whale sharks (*Rhincodon typus*) (32), cases where the monitorization of these animals highlighted the astonishing resilience of these species and the rapid speed of healing. However, the regenerative capacity of cartilage tissue in elasmobranchs has been a subject of debate in different studies. Thus, Ashhurst (33) concluded that chondrichthyans were unable to repair their cartilaginous skeleton based on an experiment involving the cutting of fin rays in dogfishes (*Scyliorhinus* spp.) and the observations made over a 26-week period. In contrast, recent findings have revealed evidence of a spontaneous mechanism of cartilage repair in response to small injuries in rays (34, 35). However, injuries involving extensive and severe damage to the skeleton of elasmobranchs have been poorly documented. The aim of this study is to provide a comprehensive description of the clinical intervention, diagnostic imaging findings, alongside the macroscopic and histological characteristics of a traumatic injury of anthropogenic origin in the scapulocoracoid cartilage of a spiny butterfly ray.

## 2 Materials and methods

**Figure 1:**
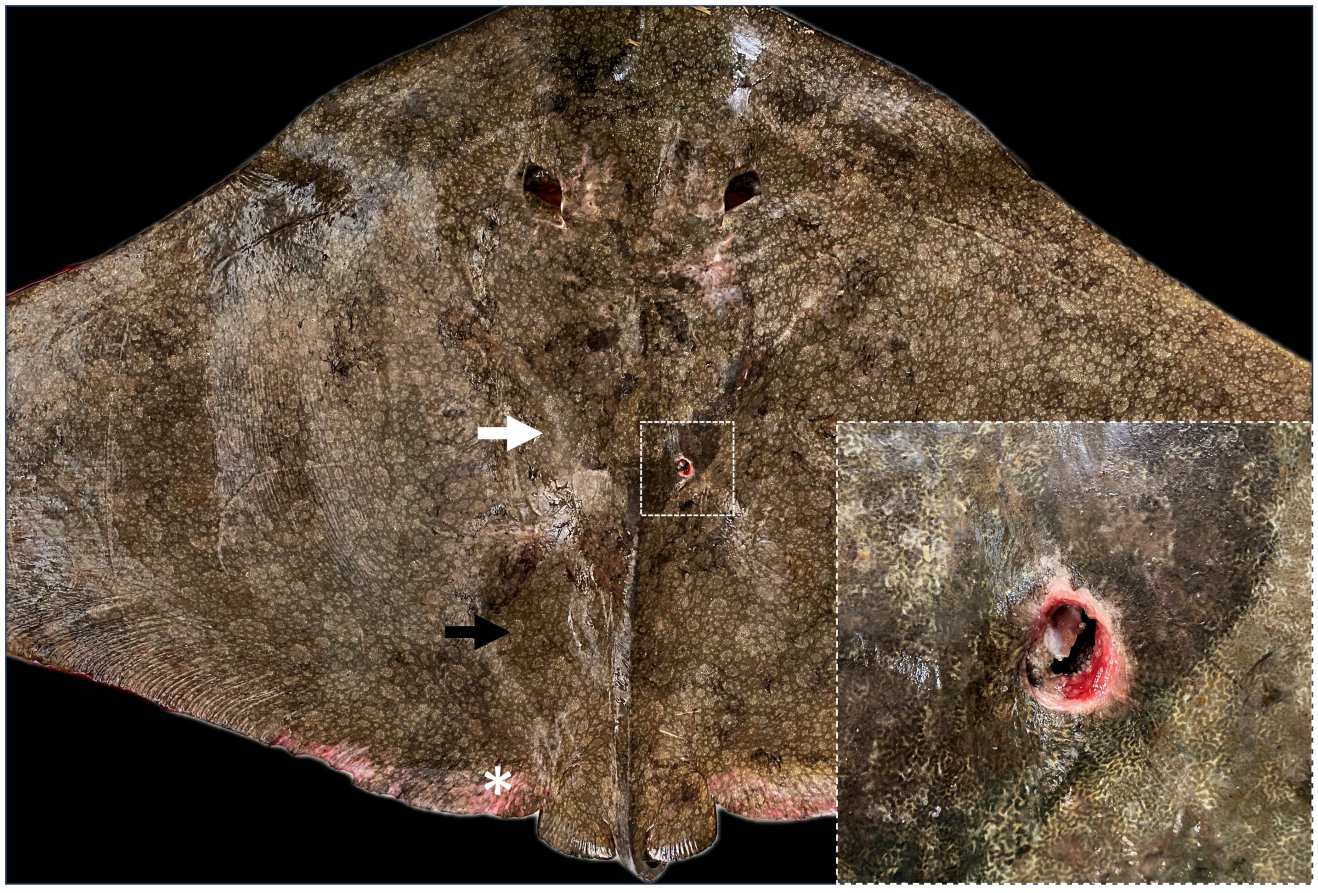
Dorsal view of the animal with the penetrating wound. The pectoral arch of the skeleton (white arrow) and the severe concavity on the dorsal coelomic surface (black arrow) are both observed as a result of the animal's cachectic state. Inset: detail of the incised-contused wound of 2 cm diameter and a depth of ~5 cm. It can be appreciated erythema, congestion, and edema at the edges of the lesion. In addition, desquamation and depigmentation could be observed in the caudal region of the dorsal surface of the pectoral fins (*).

An adult female spiny butterfly ray (*Gymnura altavela*) was spotted nearby the Castillo del Romeral wharf in Gran Canaria (Canary Islands, Spain) laying lethargic in the sandy bottom with an external circular incised-contused traumatic lesion of 2 cm diameter in the scapulocoracoid cartilage (Figure 1). It was captured and transferred to the Poema del Mar Aquarium facilities (Loro Parque Fundación, Gran Canaria) under the appropriate national and regional legal permissions and authorizations to evaluate the animals condition and consider treatment and rehabilitation options. This facility has quarantine tanks suitable for the housing of this animal, and a veterinary team specialized in the handling and health care of this elasmobranch specie. The ray weighed 32 kg, had a disc width of 179 cm and a total length of 114 cm. The animal was placed in a tank of 16.252 m^3^ with a mean water temperature of 22°C and mean values of pH and salinity of 8.1 and 36.3%, respectively.

A radiographic study was conducted to assess the affected structures and determine the depth of the incision using a portable direct digital radiography equipment (Portable X-ray Orange 9020F, Mano Medical, Taden, France) and an imaging plate measuring 42.1 × 34.4 cm (ClaroX 1417, CVM Diagnstico Veterinario S.L., Tudela, Spain). The animal was carefully transferred from the tank by several aquarists using a specialized stretcher and placed on the imaging plate, which was wrapped in plastic bags to protect it from exposure to water. Following image acquisition, the ray was immediately returned to the tank. A blood sample was collected from the pectoral fin vasculature for hematological analysis, using a 23G needle attached to a 3 mL syringe. After extraction, blood was transferred into 1.3 mL lithium heparin anticoagulant tubes (Sarstedt^^®^^ Micro Sample Tube Li-Heparin LH, Nümbrecht, Germany). Blood smears were made using the slide-to-slide technique and stained using a Diff-Quick stain (T.R.H., Maim S.L., Barcelona, Spain).

The initial treatment plan consisted in vitamin C 12.5 mg/kg, ceftazidime 25 mg/kg and dexamethasone 1 mg/kg by intramuscular injection. Despite these efforts, the animal died four days later and was transferred to the Institute of Animal Health and Food Safety (IUSA) of the ULPGC for further analysis.

A Computed Tomography (CT) study was conducted postmortem before starting the necropsy at the University Hospital of the Veterinary College. Sequential slices were acquired using a 16-slice helical CT scanner (Toshiba Astelion, Canon Medical System^Ⓡ^, Tokyo, Japan). The animal was symmetrically positioned in dorsal recumbency on the stretcher, with craniocaudal entry, and a standard clinical protocol was used (120 kVp, 50 mA, 512 × 512 acquisition matrix, 1,809 × 834 field of view, pitch of 0.94, and a gantry rotation of 1.5 s), to acquire images of a 1 mm thickness. CT images were acquired in transverse planes from cranial to caudal during dorsal recumbency. Using these transverse images, reconstructions were made in the dorsal and sagittal planes, with the images being displayed using both bone and soft tissue windows. All these images were uploaded to an image viewer (OsiriX MD v. 13.0.2, Apple, Cupertino, CA, USA) in DICOM format to perform data manipulation.

The necropsy was performed at the facilities of the Institute of Animal Health and Food Safety (IUSA) of the ULPGC, through a systematic approach and observation of external and internal organs (36). Samples from the wound and main organs were fixed in 10% neutral buffered formalin for histopathological examination. In addition, to make a comparison between normal and affected cartilage, a sample from the non-affected scapulocoracoid cartilage was taken. Cartilage tissues were placed in a histological decalcifier (Decalcifier DC2, Qpath^Ⓡ^, Fontenay-sous-bois, France) for 7 days. Formalin-fixed tissues samples were placed into cassettes and routinely processed. This included dehydration through ascending grades of alcohols, clearing in xylene and finally paraffin wax imbibition. Paraffin blocks were sectioned at 4 μm and stained with hematoxylin and eosin (H&E), periodic acid–Schiff (PAS) and Masson's trichrome (MT). The slides were mounted and examined with a light microscope (Olympus BX51, Tokyo, Japan) equipped with a camera software for DP21 (Olympus DP21, Tokyo, Japan).

## 3 Results

### 3.1 Clinical examination and hematology

The ray was kept in a quarantine tank since the arrival at the Poema del Mar Aquarium. External examination revealed a low body condition score with marked muscle wastage and severe concavity on the coelomic surfaces (Figure 1). Despite the administered treatment, the animal presented decreased responsiveness and refrained from eating during the days it was kept under human care. A differential leukocyte count was performed on the blood sample obtained, in which lymphocytes were the most abundant leukocyte with 63%, followed by 20% eosinophils or coarse eosinophilic granulocytes, 9% heterophils or fine eosinophilic granulocytes, 7% monocytes and 1% basophils. The packed cell volume (PCV) was measured and the obtained results of 15% revealed a significant decrease of 13.4% compared to an average value of 28.4% PCV gathered from previous clinical experiences for this specie (*n* = 41), working with wildlife populations in the Canary Islands (CanBio Project).

### 3.2 Radiographic and computed tomography examination

**Figure 2:**
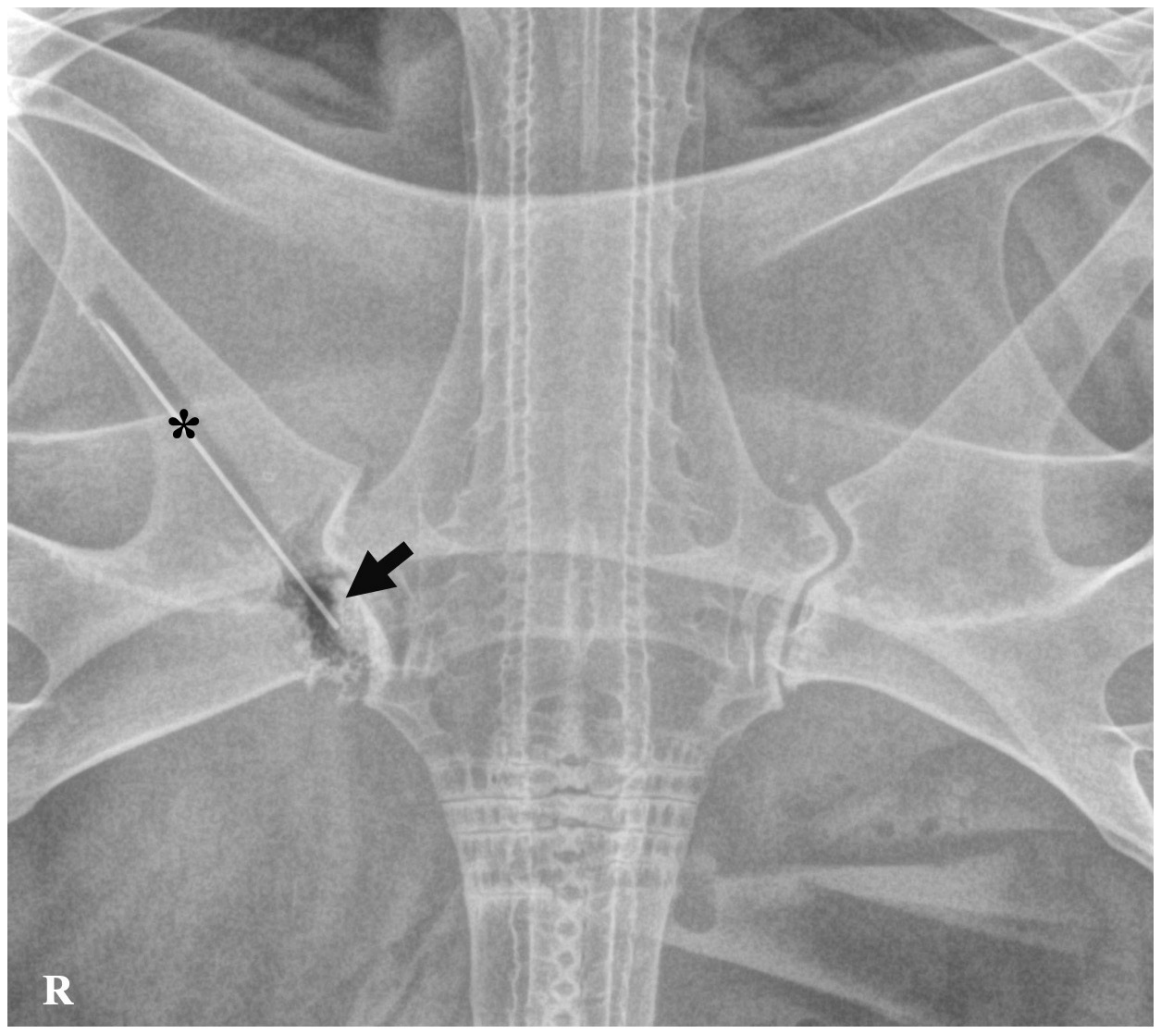
Dorsoventral radiography of the spiny butterfly ray (*Gymnura altavela*). Hypodermic needle with a cap (*) inserted into the incised-contused wound. Irregular margins with adjacent gas opacity are observed on the scapulocoracoid-synarcual joint (arrow).

One single radiography in dorsoventral projection was shot with a 0.9 × 70 mm hypodermic needle with its cap (Sterican^Ⓡ^ deep intramuscular with long bevel, B. Braun, Melsungen, Germany), introduced inside the incision to determine the depth of the wound (Figure 2). Regarding the radiographic findings, the digital radiograph revealed no affection to any vital organs, but there was severe destruction of cartilage at the level of the scapulocoracoid-synarcual joint.

**Figure 3:**
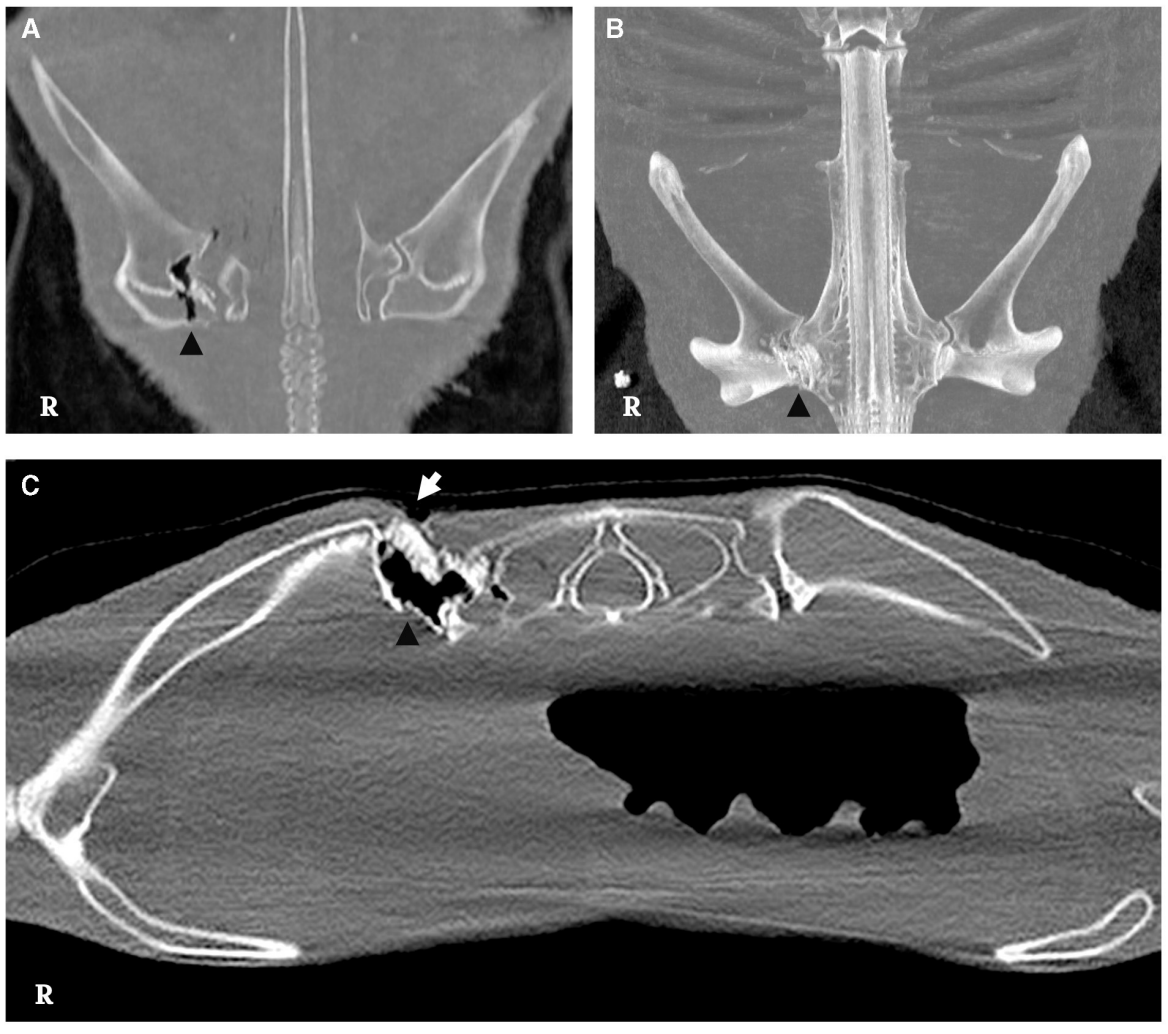
CT scan of the lesion. **(A, B)** Irregular borders are observed in the medial aspect of the scapulocoracoid cartilage with presence of slight gas attenuation (arrowhead) [bone window, dorsal multiplanar reconstruction (MPR) and dorsal maximum intensity projection 3D (MIP), respectively]. **(C)** Bone window, cross-sectional image, displaying discontinuity of the skin on the dorsal aspect of the scapulocoracoid-synarcual joint (white arrow), featuring irregular margins and gas attenuation presence (arrowhead).

CT findings unveiled a destructive lesion with irregular margins on the right side, affecting the articular surfaces that establish the pectoral arch of the synarcual cartilage with the scapulocoracoid cartilage, specifically the distal and proximal articular surfaces, as well as cranially the glenoid surface (Figures 3A–C).

### 3.3 Necropsy

#### 3.3.1 Gross pathology

**Figure 4:**
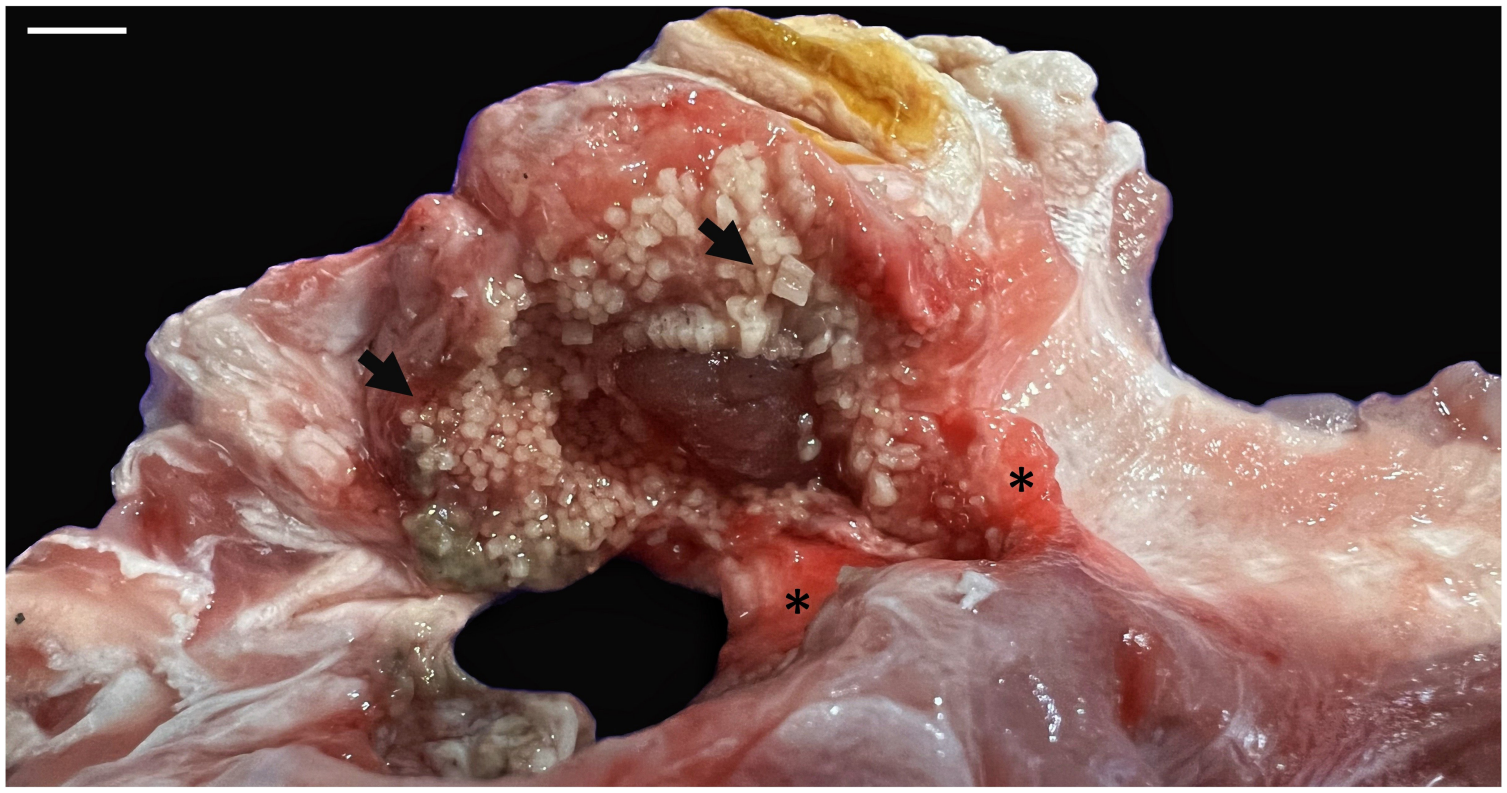
Macroscopic aspect of the lesion in the scapular process of the scapulocoracoid cartilage. Numerous square to rectangular geometric structures of hard consistency and whitish color were observed (arrows). The adjacent muscular tissue showed reddened and edematous areas (*). Scale bar = 1 cm.

In the external examination, at the level of the circular wound was observed hyperemic and edematous injured tissue and severe damage of the cartilage at the joint below (inset Figure 1). The lesion reached a depth of ~5 cm, affecting the scapular process of the scapulocoracoid cartilage and the articular surface of the synarcual. Once opened, this area, showed numerous square and rectangular geometric structures of hard consistency and whitish color (Figure 4). The adjacent muscular tissue showed reddened and edematous zones.

**Figure 5:**
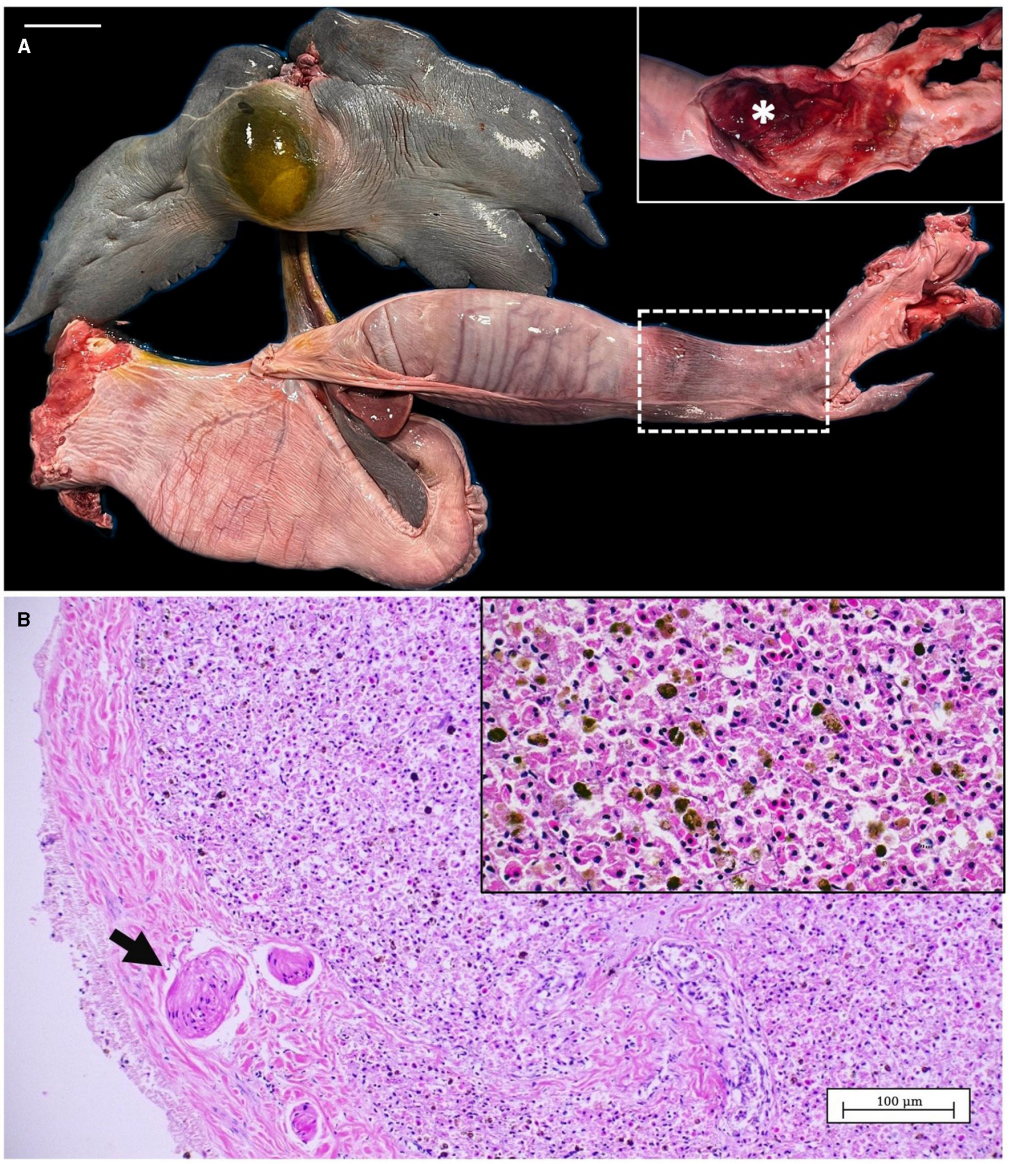
**(A)** Liver and gastrointestinal system from spiny butterfly ray. Inset: Detail of the colon, once opened, with moderate hemorrhage (*). **(B)** Microscopic view of the liver parenchyma showing concentric smooth muscle fibers in the liver capsule (arrow). Inset: magnification of the liver showing a low amount of intracellular lipid drops in the hepatocytes and abundant accumulation of pigments. Scale bar = 5 cm.

In the internal examination, the liver displayed a marked reduction in size (727.5 g) with dark gray coloration, rounded edges of hepatic lobes, readily visible capsule, and a severe distension of the gallbladder (Figure 5A). Also, an empty digestive tract and moderate hemorrhage in the colon was observed (Figure 5A, inset). Body and liver weight, hepatosomatic index and gross appearance of the liver were compared with necropsy reports of other spiny butterfly rays studied by our research group. The hepatosomatic index of this animal was 2.51 representing a 32.89% decrease compared to the average of measured animals (3.74) (Supplementary material). Microscopically, the liver showed low storage of lipid drops in the hepatocytes when it is compared with those observed from livers of non-cachectic spiny butterfly rays (Supplementary Figure 1). Large accumulations of pigments diffusely distributed were also observed in the liver parenchyma, as well as the presence of aggregates of concentric smooth muscle fibers in the liver capsule (Figure 5B).

#### 3.3.2 Histopathology

**Figure 6:**
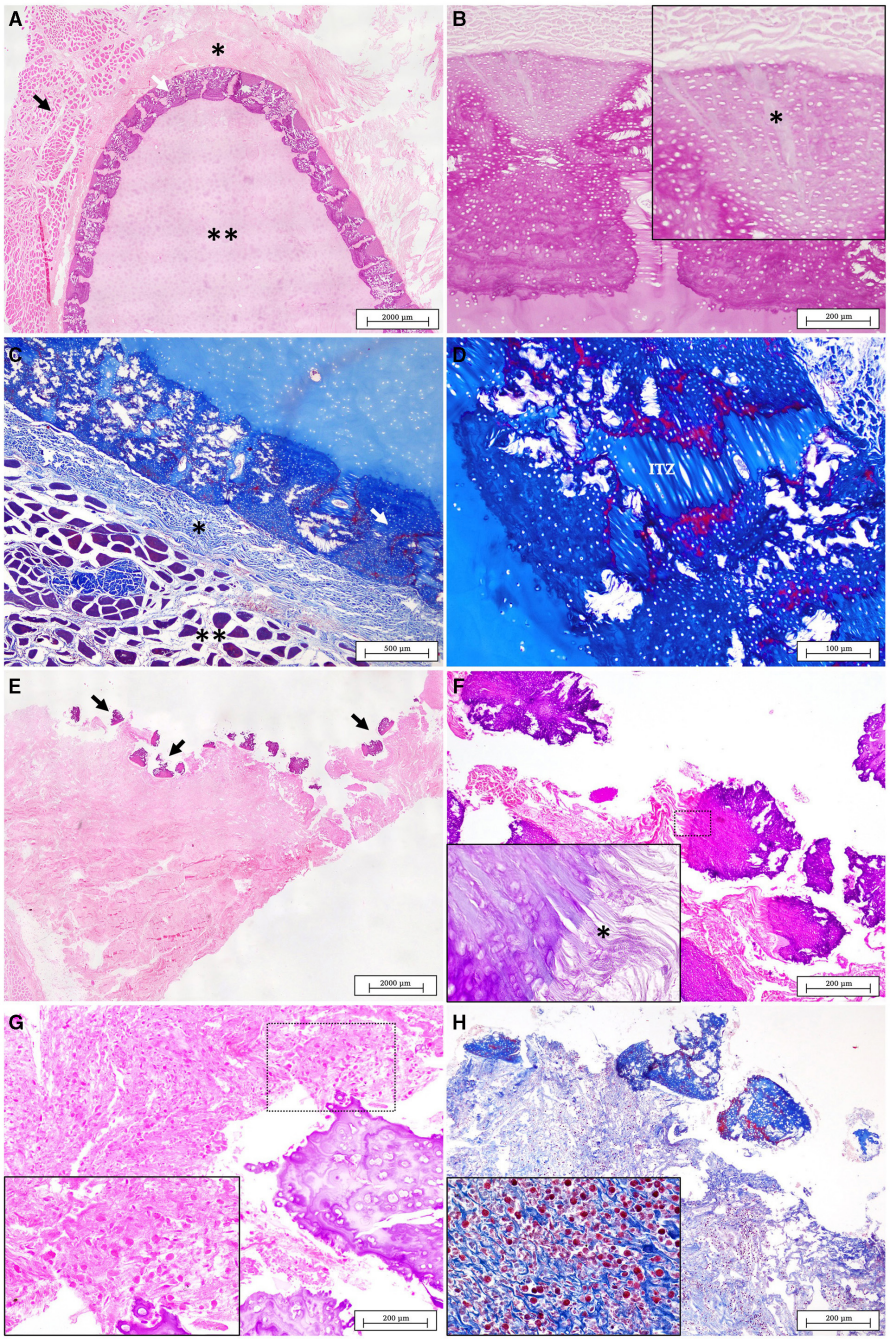
**(A)** Normal structure of healthy cartilage displaying four distinct layers, from the outer to the inner: the muscle layer (black arrow), the perichondrium (*), tesserae layer (white arrow) and the unmineralized cartilage core (**) (H&E). **(B)** Normal tesserae. Sharpey's fibers from the perichondrium are observed penetrating in the cap zone of the tesserae (*) (PAS). **(C)** Normal structure of the healthy cartilage stained with MT. The muscle layer is evident in red (**) while the perichondrium (*) and the mineralized tesserae layer (white arrow) are seen in intense blue (MT). **(D)** A detail of the fibrous intertesserae zone (ITZ) stained in blue with MT. The edges of each tesserae piece are shown marked in red probably due to different types and degrees of maturation of collagen fibers (MT). **(E)** Lesion in the scapulocoracoid cartilage of the affected animal. The injured area is characterized by the disorganization of the tesserae layer (arrows), with the presence of broken tesserae pieces over an abundant fibrous connective tissue (H&E). **(F)** Detailed of fractured pieces of tesserae displaced from the adjacent perichondrium (H&E). (Inset) fraying of the Sharpey's fibers were observed in each piece of tesserae (*) (PAS). **(G)** The fibrous tissue below the broken tesserae layer appears increased and infiltrated by an abundant inflammatory cell population (H&E). **(H)** The MT stain revealed the absence of the muscle layer while it helps to identify granulocytes as the main cells of the inflammatory infiltrate (MT).

Within this section we report the comparison of the histological features of the normal cartilage with those of affected tissue. The Figure 6A show the normal structure of the spiny butterfly ray scapulocoracoid cartilage. An outer calcified ring of polygonal tiles (tesserae layer) is observed between the unmineralized cartilaginous core and the fibrous perichondrium. In Figure 6B a magnification of the tesserae layer is shown with the detail of thick Sharpey's fibers penetrating from the perichondrium into the cap zone of each tesserae pieces.

In the normal structure using Masson's Trichrome (MT) staining, the perichondrium layer (connective tissue), is usually marked in blue while the muscle layer can be observed stained in red (Figure 6C). On the peripheral edges of normal tesserae pieces, clear differentiated areas are stained also in red in contrast with an intense blue of the fibrous intertesserae zones (ITZ). According to what have been previously published by other authors, these changes could be related with different collagen compositions (35, 37) (Figure 6D).

The cartilage affected by the traumatic lesion showed the disorganization of the pieces of tesserae, separated from the unmineralized core, and displaced from the adjacent perichondrium (Figure 6E). In the injured area of cartilage, fragmentation and fraying of the Sharpey's fibers were observed in each piece of tesserae leading to the rupture and damage of the perichondrium (Figure 6F). The injured area showed the lack of the normal layers of the different tissues surrounding the joint (Figures 6G, H). The articular muscles are not evident anymore and they have been replaced, in the damaged area, by an abundant fibrous tissue with an intense inflammatory cell population, mostly composed by granulocytes and mononuclear cells (insets in Figures 6G, H).

## 4 Discussion

Anthropogenic causes of death in elasmobranchs are frequently observed. Overfishing is one of the biggest threats to these species, that together with climate change and habitat degradation have caused a worldwide decline in sharks and rays populations (2, 38, 39). Bycatch is the main cause, particularly in pelagic longline fisheries (40), but also illegal trading or even ship collisions are responsible of large numbers of deaths every year (41, 42). These interactions between human activities and elasmobranchs can lead to a wide range of injuries. The main pathologies resulting from these fishing interactions are hypoxia and trauma, including blunt-force trauma, penetrating trauma from different fishing instruments and the wounds arising from hooks to the oral cavity, digestive tract, and gills (43).

This study describes the clinical presentation, diagnostic imaging evaluation and anatomopathological features of a traumatic lesion in the scapulocoracoid and synarcual cartilages in a spiny butterfly ray. The topography of the incised-contused wound, located dorsally at the level of the right scapulocoracoid cartilage, coupled with the macroscopic characteristics of the lesion and the findings from the CT scan, suggested an anthropogenic origin with some fishing instrument (pike pole, harpoon, or similar). This penetrating wound impacted the juncture of the scapulocoracoid and synarcual cartilages, resulting in significant movement impairment. Other pathological findings included a decreased liver size, hemorrhagic enteritis, and an empty digestive tract. These observations suggest a chronic process of debilitation and a potential compromise of predatory behaviors that could ultimately lead to the death of the animal.

Due to the animal's penetrating wound, pharmacological treatment was immediately initiated to aid recovery upon arrival at the Poema del Mar Aquarium facilities. There is little data on pharmacokinetics and pharmacodynamics for most drugs used in elasmobranch medicine, particularly in some species, so clinicians use treatments that are often extrapolated from taxonomically close species or are based on previous clinical experience (44). Since septicemias are common in batoids with non-healing wounds (45), ceftazidime was chosen as antibiotic coverage. Dexamethasone was administered to control inflammation associated with the wound. Vitamin C was administered due to its role as a cofactor involved in collagen and cartilage synthesis, which is commonly employed as nutritional supplementation in elasmobranchs kept under human care (46). In terms of hematological parameters, although elasmobranchs can have a low hematocrit in comparison to other species (47, 48), our result of 15% was lower compared to unpublished data obtained for the same species.

The marked reduction in liver size suggested that the animal was undergoing a chronic debilitation process, likely resulting due to difficulties in moving and feeding (44, 45, 49). The liver in elasmobranchs is the primary reservoir of triglycerides and the principal energy source when affected by periods with absence of intake; a decrease in its size may be related to prolonged fasting periods, high energy demand or stressful circumstances (49). In agreement with Neyrão et al. (50) histopathological analysis of the liver revealed characteristics consistent with prolonged fasting, such as diminished lipid content and increased number of melanomacrophages. Similarly, the gallbladder was severely distended, which could also be related to a long period of feeding cessation (51).

The hemorrhages observed in the region of the colon are associated with an inflammatory process of enteritis. This lesion can be frequently observed secondary to other many pathologies in elasmobranchs, such as septicemia or parasitic infections (52–55) and it could also contribute to the overall poor health status observed in this butterfly ray.

The radiographic examination of elasmobranchs is limited due to the lower density of internal structures when compared to other species of animals, yet they offer remarkable detail for the assessment of the cartilaginous skeleton (56). In this work, the utilization of radiography allowed us to inspect the depth of the incision and the affected structures while the animal was alive. Due to the significant dorsoventral flattening of the spiny butterfly ray, alternative radiographic projections with conventional radiographic techniques were impractical for obtaining more detailed information of the lesion. However, this issue was addressed post-mortem by conducting a CT study. The application of this type of advanced diagnostic imaging techniques, provides a more accurate and comprehensive evaluation of lesions in zoological animals (57). The results showed lysis of the right scapulocoracoid cartilage, affecting the articular surfaces that form the pectoral arch connection between the synarcual and scapulocoracoid cartilages. In our best knowledge this is the first CT description of a traumatic lesion of the skeleton in a butterfly ray.

The skeleton of elasmobranchs is composed of cartilaginous tissue consisting of a mineralized layer constituted by minute, polygonal tiles called tesserae that lies in between the cartilage core and the perichondrium (58). In the present study, multiple whitish, disordered geometric structures corresponding to disorganized pieces of tesserae were observed, macroscopically, in the injured cartilage. Likewise, the disruption of the tesserae pieces was evident in the microscopic study. To our knowledge, this is the first complete anatomopathological description of this lesion in the skeleton of a spiny butterfly ray.

There has been controversy in recent decades about cartilage regeneration in elasmobranchs. Ashhurst (33), following a 26-week experiment involving cutting the fin rays cartilages of dogfishes, concluded that, chondrichthyans, were unable to repair their cartilaginous skeleton. Although cartilage-like tissue did develop by 12 weeks, it exhibited poor vascularization and failed to integrate with the injured tissue. However, a more recent study by Seidel et al. (34) in sharks and rays described the aberrant development of mineralized cartilage-like tissue, referred to as endophytic masses (EPMs), exhibiting ultrastructural and chemical characteristics distinct from tesserae. While the formation of EPMs was considered a potential form of attempted cartilage repair, the authors concluded that the most likely cause was a local breakdown of CaP mineralization inhibition processes. Marconi et al. (35) reported, for the first time, the presence of cartilage progenitor cells and chondrogenesis in adults of the little skate (*Leucoraja erinacea*), demonstrating the ability to spontaneously repair injured cartilage. The repair tissue shared characteristics with normal tissue, comprising type II collagen and seamlessly integrating with adjacent tissue to rectify irregularities. Notably, their study involved surgical incisions of < 2 cm and the removal of cartilage with a 4 mm biopsy punch, followed by wound suturing and postoperative antibiotherapy. In this study, we observed a chronic injury characterized by the disorganization of the tesserae layer of the cartilage, with an inflammatory reaction and extensive edema and hemorrhage. The severity of the trauma precludes cartilage regeneration, leading to a chronic process that could impair the animal's mobility and complicates feeding. Despite different traumatic injuries have been studied in sharks and rays (16–24), there are scarce detailed descriptions of the pathological features of these lesions on elasmobranchs. To our best knowledge, this study represents the first comprehensive description of the diagnostic imaging findings and the macroscopic and histological characteristics of a traumatic injury of anthropogenic origin in the skeleton of a spiny butterfly ray.

## 5 Conclusions

This study reports the first comprehensive description of skeleton trauma in a spiny butterfly ray, including the clinical presentation, diagnostic imaging and the anatomopathological features. These three points of view lead to the conclusion that, the incised-contused wound had an anthropogenic origin, probably, by some kind of fishing instrument (pike pole, harpoon, or similar).

Although the animal was found alive and kept in a quarantine tank, under veterinary healthcare and treatments, unfortunately, the establishment of a chronic pathological process with a marked intake reduction, movement reluctancy and the absence or low regenerative processes, on the affected tissues, produced an overall debilitation status that, eventually, might cause the death of the animal.

Together with the clinical signs and administered treatment, our work provides the assessment of hematological parameters on the dying animal, resulting on a notable hematocrit reduction of the 13.4%, when compared with the average of healthy wildlife spiny butterfly ray populations. Furthermore, the clinical evaluation provides, for the first time, a CT study of a critical traumatic lesion, of the right scapulocoracoid cartilage and associated articular surfaces, in this species. The anatomopathological findings also confirmed that the animal was undergoing a chronic debilitation process. The specimen showed a marked reduction of the 32.89% in the hepatosomatic index, when compared to the average value of non-cachectic female spiny butterfly rays. Histological findings demonstrated a remarkable reduction of lipid drops in the cytoplasm of the hepatocytes.

Our work showed, for the first time, the macroscopic and microscopic description of the non-regenerative disorganization of the tesserae layer, and adjacent tissues, of the scapulocoracoid cartilage, due to a traumatic lesion. The main characteristics were the presence of whitish square to rectangular geometric pieces of tesserae, separated among them and torn from the cartilaginous core. Histologically, some of these pieces kept their connection of the Sharpey's fibers with the perichondrium demonstrating its strong resistance. However, due to the traumatic impact, the surrounding soft tissues showed edema, hemorrhages, and a persistent inflammatory process, composed mainly of granulocytes and fibrous connective tissue. This reaction failed to produce regeneration of the cartilage and the soft tissues around the open wound including the epidermis, dermis, and the periarticular fibrous and muscular tissues.

Finally, there is still an important lack of knowledge on the clinical and diagnostic approaches to elasmobranch species, many of which are affected by anthropogenic impacts and in severe risk of extinction. Our work contributes to reduce this gap and facilitate to the scientific communities new resources to conduct more specific clinical and pathological diagnosis and, therefore, path the way to improve treatments and develop more specific conservation and preservation management plans.

## Data Availability

The raw data supporting the conclusions of this article will be made available by the authors, without undue reservation.
