# Supplementary material for: Pathological study of a traumatic anthropogenic injury in the skeleton of a spiny butterfly ray (Gymnura altavela)
Source: Front Vet Sci. 2024 Oct 24;11:1452659. doi: 10.3389/fvets.2024.1452659 (PMC11540817; doi:10.3389/fvets.2024.1452659)
Supplement: Supplementary file 1 [file Data_Sheet_1.pdf]

Supplementary Material

1 TABLE S1

Table S1. Data obtained from spiny butterfly rays (*Gymnura altavela*) necropsied at the Fish Pathology Unit of the Institute of Animal Health and Food Safety of the University of Las Palmas de Gran Canaria (ND: not determined). The hepatosomatic index (HSI) is the ratio of liver weight to total body weight. The mean HSI in non-cachectic spiny butterfly rays is 3.74.

**Name**	**Sex**	**Disc width** (cm)	**Body weight** (kg)	**Liver weight** (kg)	**HSI**	**Diagnosis**
974/22	Female	154.8	29.5	0.92	3.11	ND
1087/22	Female	–	12.6	0.51	4.04	ND
1880/22	Female	110.7	32.1	1.11	3.46	Anthropogenic: linear cuts and multifocal hemorrhages in fins.
171/23	Female	179.0	29.0	0.73	2.51	Anthropogenic: incised-contused wound in the right scapulo-coracoid cartilage
102/24	Female	163.0	28.0	1.22	4.34	Non-anthropogenic: chronic parasitic enteritis

2 SUPPLEMENTARY FIGURE 1

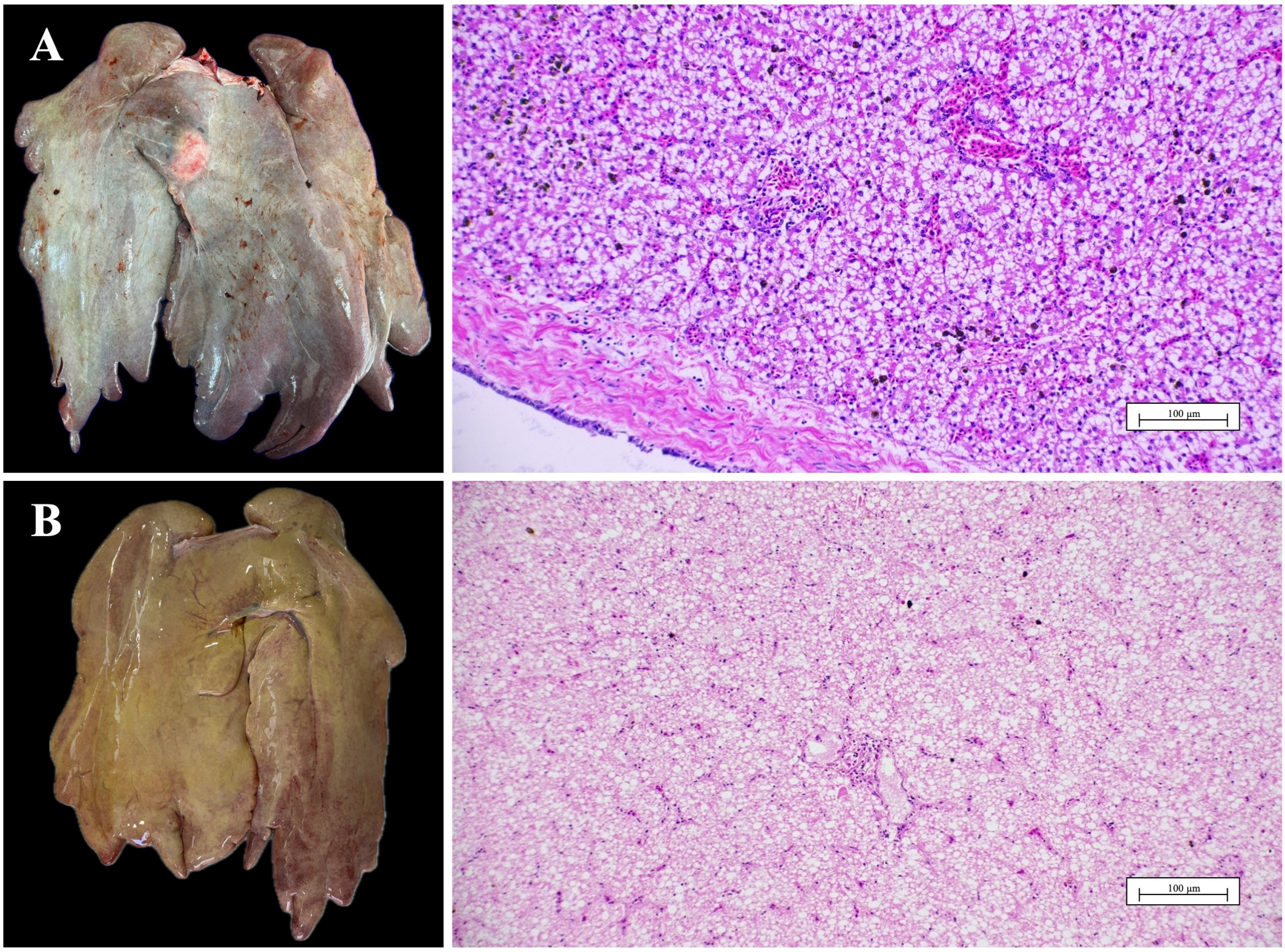

Figure 1. Macroscopic and microscopic comparison of livers of spiny butterfly rays (*Gymnura altavela*). (**A**) Liver from a spiny butterfly ray with a poor body condition. Macroscopically, a marked reduction in size, dark gray coloration, rounded edges of hepatic lobes, readily visible capsule and distension of the gallbladder were the main findings. Microscopically, low storage of lipid drops in the hepatocytes, increased number of melanomacrophages and thickening of the capsule were observed. (**B**) Liver from a spiny butterfly ray with good body condition. Macroscopically, the liver had a yellowish colour. Microscopically, a higher content of lipid drops and lower amount of melanomacrophages are observed.
